# Hand-Schüller-Christian syndrome combined with empty sella syndrome: A case report and literature review

**DOI:** 10.1097/MD.0000000000033216

**Published:** 2023-03-10

**Authors:** Wei Ji, Xiaoyang Chen

**Affiliations:** a The Second Clinical College of Fujian Medical University, Quanzhou City, China; b Department of Respiratory and Critical Care Medicine, the Second Affiliated Hospital of Fujian Medical University, Quanzhou City, China.

**Keywords:** empty sella syndrome, Hand-Schüller-Christian syndrome, Langerhans cell histiocytosis, respiratory failure, severe pneumonia

## Abstract

Hand-Schüller-Christian syndrome (HCS) is a rare disease with little clinical awareness, but the condition is more dangerous, and it combines with empty sella syndrome (ESS) which is extremely rare.

**Presentation:**

A 26-year-old male patient who had proptosis, headaches, and diabetes insipidus for more than 10 years, and chronic cough and wheeze for 8 years presented to our hospital due to an abrupt onset of chest pain for 2 days.

**Diagnosis:**

Hand-Schüller-Christian syndrome is diagnosed based on the typical clinical manifestations of diabetes insipidus and bilateral proptosis, magnetic resonance imaging (MRI) pituitary imaging and pathology. Empty sella syndrome is diagnosed based on hormonal indicators, clinical manifestations and MRI pituitary scan results. Type 1 respiratory failure and severe pneumonia can be diagnosed based on the results of clinical examination, chest imaging (including chest x-ray and computed tomography), pathology and blood gas analysis. Left pneumothorax can be diagnosed with chest imaging.

**Interventions:**

“Meropenem and Cefdinir” were given for antimicrobrial coverage, “Desmopressin acetate” for anti-diuretic treatment, “Forcodine” for cough relief, “Ambroxol and acetylcysteine” for phlegm reduction, and continuous closed chest drainage was performed.

**Outcomes:**

The patient discharged after cough, wheezing, headache and other symptoms improved, and vital signs were stable. The patient has been followed up once a month for 17 months ongoing after discharge. At present, symptoms such as cough, sputum, and wheezing have improved considerably, and the mMRC score of dyspnea is 2 points. The reexamination of the chest X-ray shows that the absorption of lung exudates is better than before, and there is no recurrence of pneumothorax.

**Lessons:**

Consider whether isolated diabetic insipidus is related to HSC, and if so, conduct an MRI, a biopsy, and other examinations as soon as possible.

## 1. Introduction

Hand-Schüller-Christian syndrome (HSC) was first proposed by Lichtenstein in 1953, commonly known as Langerhans cell histiocytosis (LCH).^[1]^ HSC is a group of rare disorders of uncertain etiology, with bone damage, diabetes insipidus, and proptosis as its hallmark symptoms.^[2]^ In 1968, Kaufman made the first report about empty sella syndrome (ESS). The subarachnoid space protrudes into the sella under the pressure of the cerebrospinal fluid, causing the sella to enlarge and the pituitary gland to compress and deform as a result of coloboma or absence of the diaphragm sella turcica, or atrophy of the pituitary gland.^[3,4]^ Symptoms of ESS include headaches, vision and visual field disorders, endocrine dysfunction, amongst others.^[4]^

HSC and ESS are rarely reported in domestic and foreign literature, especially HSC. Therefore, clinical awareness is not high. We report a case of Hand-Schüller-Christian Syndrome combined with ESS in a patient with severe pneumonia and respiratory failure, aiming to improve the understanding of the combination of HSC and ESS to reduce the rate of misdiagnosis and missed diagnosis.

## 2. Case report

A 26-year-old male patient who had been experiencing an abrupt onset of chest pain for 2 days went to the emergency department of our hospital was admitted to the respiratory medicine department for treatment after a computed tomography scan revealed a pneumothorax. During the course of treatment, it was discovered that the patient had developed a thirst for more than 10 years without any apparent explanation and that it persisted even after drinking more than 5 L of water every day. Over the next few years, the patient developed bilateral proptosis, headaches, a cough, and wheezing, and suffered 2 epileptic seizures. The patient was eventually identified with “histiocytosis” through a tissue biopsy after several outpatient medical appointments.

**Figure 1.**
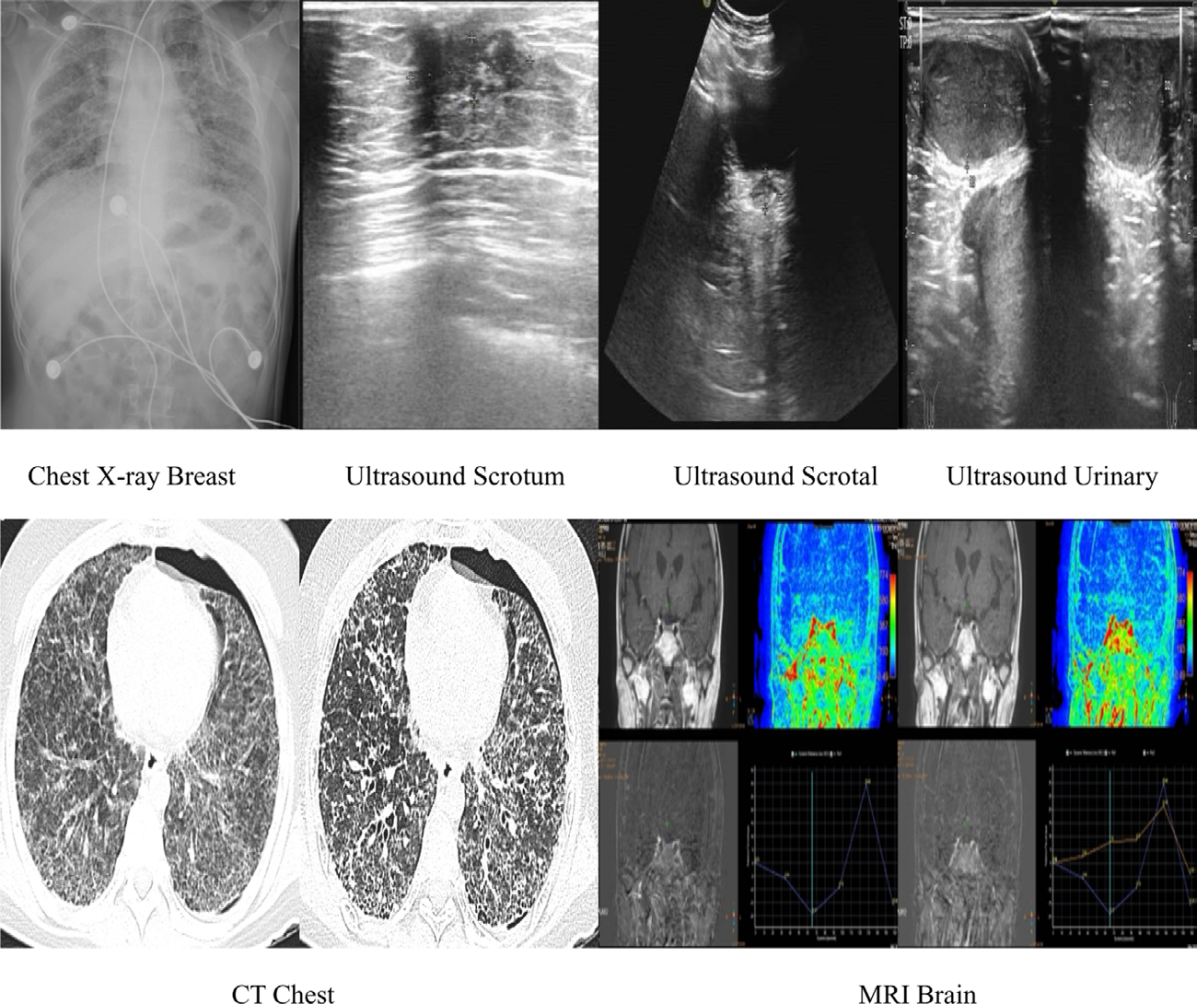
Ultrasound scrotum, ultrasound scrotal, ultrasound urinary, chest X-ray breast, CT chest, and MRI brain. CT = computed tomography, MRI = magnetic resonance imaging.

On admission the patient’s blood pressure was 126/77 mm Hg, temperature was 36.3°C, heart rate was 94 beats/min, and respiratory was 30 beats/min. He was a short and odd-looking patient who had poor nutrition and mental retardation. We did not palpate a pleural rub, nor abnormalities in percussion of the chest wall. There were sporadic moist rales audible in both lungs, with reduced breath sounds in the left lung. There were no audible signs of dry rales or pleural friction rub. No precordial prominence was present, and the apical beat was situated in the fifth intercostal space 0.5 cm medial to the left mid-clavicular line. There is no palpable tremor and pericardial friction. The patient has normal cardiac boundary, and has regular heart rhythm and normal heart sounds. Laboratory results showed carbon dioxide partial pressure was 46 mm Hg, oxygen partial pressure was 57 mm Hg, and oxygenation index was 196 mm Hg. Blood examination showed a white blood cell count of 21.18 × 10^9^/L, neutrophils of 18.48 × 10^9^/L, and a hemoglobin of 134 g/L. Fasting blood glucose was 3.45 mmol/L, and 2-hour postprandial blood glucose was 5.63 mmol/L. Fasting c-peptide and 2-hour postprandial c-peptide were normal. Cortisol was normal at different periods. Sputum analysis, bacterial sputum smear, fungal sputum smear, tuberculosis sputum smear, and vasculitis-related autoantibody detection were all unremarkable. Adrenocorticotropic hormone was 28.25 pg/mL, parathyroid hormone was 45.97 pg/mL, human growth hormone was 1 ng/mL, luteinizing hormone was 0.974 mIU/mL, follicle-stimulating hormone was 1.34 mIU/mL, progesterone was 0.103 ng/mL, prolactin was 6.22 ng/mL, testosterone was 0.396 ng/mL, estradiol was 7.42 pg/mL. On ultrasound (Fig. 1), the patient was found to have breast development, testicular atrophy, bladder wall thickening and irregular-jagged alterations. On computed tomography and X-ray (Fig. 1), the patient was found to have left pneumothorax and bilateral interstitial lung lesions. On magnetic resonance imaging (Fig. 1), the patient’s sella is replaced by a liquid signal foci, the anterior pituitary tissue is thin, the posterior pituitary is not shown, and the slope is thickened showing a ground glass shape.

We managed the patient with meropenem and cefdinir, desmopressin acetate, continuous closed drainage of the thoracic cavity, fluid support, and other treatments. The patient was discharged after the discomfort of wheezing, coughing and sputum subsided. Following discharge, the patient underwent routine followed-up once a month for 17 months. At present, the symptoms of cough, expectoration, wheezing, and other symptoms have all improved significantly, and the mMRC score for dyspnea is 2 points. The review chest X-ray reveals that there has been no recurrence of pneumothorax, and improvement of the pleural effusion. (The patient has provided informed consent for publication of the case.)

## 3. Discussion

Hand-Schüller-Christian Syndrome is a very rare disease that can strike anyone at any age, with a peak incidence at 1 to 3 years of age. It affects more males than females, and has an incidence of 4 to 8 per million in children, but the incidence in adults is just 1 to 2 in a million.^[5,6]^ Many patients are unable to receive the proper diagnosis and treatment because of the low incidence, limited clinical awareness, and high missed diagnostic rate of this condition.^[6]^ HSC frequently involves a number of organs and systems, although it can also involve only one, and the most vulnerable tissues include lymph nodes, bone, skin, and the lung. The most common symptom of HSC is solitary pulmonary LCH.^[1,6]^ The patient’s age and the level of lesion involvement affect the prognosis, tending to be worse in younger patients with a multiple sites of disease.^[7]^ Histiocytosis that occurs in infancy often has an acute onset and rapid progression. There are various degrees of damage, and the chances of survival through adulthood are quite slim. Death usually occurs more than a few weeks to 2 years after the first sign of illness.^[7,8]^ The etiology of HSC is currently unknown, though current literature suggests genetic mutation, tumor microenvironment, and viral infection may play roles.^[9]^ There is debate regarding whether harm is caused by the dendritic cells’ malignant mutation or the inflammatory infiltration, but typical clinical manifestations and imaging findings aid diagnosis.^[9,10]^ The gold-standard for diagnosis is a combination of clinical presentation and pathological demonstration of LCH, but clear imaging findings can also aid in diagnosis.^[5,6,8,11]^ The patient has been treated with individual strategy and obtain successful outcomes.^[12]^

ESS was once perceived by the general public as a rare disease,^[13,14]^ but thanks to ongoing advances in medical technology, its incidence in population imaging censuses have increased from 5.5% to 35%. ESS can be divided into primary and secondary forms.^[12,13]^ The early stages of ESS are frequently asymptomatic, though a small percentage of individuals experience unusual symptoms such as headache, visual impairment, cerebrospinal fluid rhinorrhea, and endocrine disorders. Though pathogenesis of ESS is still unclear, prevailing theories^[12,15]^ include abnormal sella development, continuous or intermittent increase in cerebrospinal fluid pressure, obesity, and genetic factors. The standard for ESS diagnosis is undoubtedly imaging.^[13,15]^ Treatment is not necessary for asymptomatic ESS, but continuous follow-up and treatment is necessary to monitor for symptom development.

In this case, we report a rare combination of HSC and ESS that has not previously been reported in domestic and foreign literature. The patient suffered from HCS at an early age. HCS lesions can affect the bones of the head, leading to the loss of the integrity of the diaphragm sella turcica. Subarachnoid herniation into the sella may cause ESS after the anatomical structure changes. Furthermore, the diabetes insipidus in this case has not been cured or considerably controlled during many years of treatment. The long-term presence of diabetes insipidus may be explained by the coexistence of HCS lesions involving the head and ESS, which cause endocrine disorders and aberrant hormone secretion. A definite association has been noted in the literature in recent years between ESS and the HSC that induced diabetes insipidus in patients as well as between interstitial lung disease and HSC.^[16,17]^ However, because there are no other secondary causes, ESS may also be primary. Although the symptoms and signs of diabetes insipidus and exophthalmos may be caused by HSC and ESS, the coexistence of HCS and ESS may not be relevant.

In general, HCS is considered a pediatric disorder, but it can also occur in adults, and it is often challenging to trace the cause in isolated diabetes insipidus.^[2]^ HCS is a rare disease with little clinical awareness, but the condition is dangerous. Missed diagnosis or misdiagnosis of this disease will delay diagnosis and treatment and possibly even have an adverse effect on prognosis. Therefore, it is crucial for clinicians to determine whether diabetes insipidus is a part of HSC. Early diagnosis of the patient’s illness and knowledge of the relationships between diseases will make it possible to treat the patient more effectively and at an earlier stage. The prognosis is excellent if HCS just affects the skin, and in some patients the condition is self-limiting. However the condition becomes extremely hazardous if it affects other systems. Death may occur within a few days of illness onset, usually from secondary systemic infections or organ failure. The disease affected the lungs in this example, but due to prompt identification and treatment, the prognosis was positive.

## Author contributions

**Writing – original draft:** Wei Ji.

**Writing – review & editing:** Wei Ji, Xiaoyang Chen.
